# Significance of postoperative adjuvant chemotherapy with an oxaliplatin-based regimen after simultaneous curative resection for colorectal cancer and synchronous colorectal liver metastasis: a propensity score matching analysis

**DOI:** 10.1186/s12893-021-01193-4

**Published:** 2021-04-09

**Authors:** Kiichi Sugimoto, Kazuhiro Sakamoto, Yuki Ii, Kota Amemiya, Hiroyuki Sugo, Tomoaki Ito, Shinya Munakata, Makoto Takahashi, Yutaka Kojima, Yuichi Tomiki, Koichi Sato, Akio Saiura, Seiji Kawasaki

**Affiliations:** 1 Department of Coloproctological Surgery, Juntendo University Faculty of Medicine, 2-1-1 Hongo, Bunkyo-ku, Tokyo, 113-8421 Japan; 2 Department of General Surgery, Juntendo University Nerima Hospital, Tokyo, Japan; 3 Department of Surgery, Juntendo University Shizuoka Hospital, Shizuoka, Japan; 4 Department of Hepatobiliary-Pancreatic Surgery, Juntendo University Faculty of Medicine, Tokyo, Japan

**Keywords:** Colorectal cancer, Synchronous liver metastasis, Postoperative adjuvant chemotherapy, Oxaliplatin-based regimen, Propensity score matching analysis

## Abstract

**Background:**

Expansion of the indication for liver resection and new regimens for systemic chemotherapy have improved postoperative outcomes for synchronous colorectal liver metastases (CRLM). However, such cases can still have a high recurrence rate, even after curative resection. Therefore, there is a need for postoperative adjuvant chemotherapy (POAC) after liver resection in patients with CRLM. There are few studies of the efficacy of POAC with an oxaliplatin-based regimen after simultaneous resection for colorectal cancer and CRLM with curative intent. The goal of the study was to compare POAC with oxaliplatin-based and fluoropyrimidine regimens using propensity score (PS) matching analysis.

**Methods:**

The subjects were 94 patients who received POAC after simultaneous resection for colorectal cancer and synchronous CRLM, and were enrolled retrospectively. The patients were placed in a L-OHP (+) group (POAC with an oxaliplatin-based regimen, n = 47) and a L-OHP (−) group (POAC with a fluoropyrimidine regimen, n = 47). Recurrence-free (RFS), cancer-specific (CSS), unresectable recurrence-free (URRFS), remnant liver recurrence-free (RLRFS), and extrahepatic recurrence-free (EHRFS) survival were analyzed.

**Results:**

Before PS matching, the L-OHP (+) and (−) groups had no significant differences in RFS, CSS, URRFS, RLRFS, and EHRFS. Univariate analysis indicated significant differences in age, preoperative serum CEA (≤ 30.0 ng/mL/ > 30.0 ng/mL), differentiation of primary tumor (differentiated/undifferentiated), T classification (T1–3/T4), number of hepatic lesions and maximum diameter of the hepatic lesion between the L-OHP (+) and (−) groups. After PS matching using these confounders, RFS was significantly better among patients in the L-OHP (+) group compared with the L-OHP (−) group (HR 0.40, 95% CI 0.17–0.96, p = 0.04). In addition, there was a trend towards better RLRFS among patients in the L-OHP (+) group compared with the L-OHP (−) group (HR 0.42, 95% CI 0.17–1.02, p = 0.055). However, there were no significant differences in CSS, URRFS and EHRFS between the L-OHP (+) and (−) groups.

**Conclusions:**

PS matching analysis demonstrated the efficacy of POAC with an oxaliplatin-based regimen in RFS and RLRFS.

## Background

Colorectal cancer is a global cause of death and an increasingly common disease in Japan [1, 2]. Expansion of the indication for liver resection and new regimens for systemic chemotherapy have altered therapeutic strategies and improved postoperative outcomes for synchronous colorectal liver metastases (CRLM) [3]. In cases with resectable CRLM, treatment with liver resection is now thought to be the best strategy [4–6]. However, such cases can still have a high recurrence rate, even after curative resection [7]. Therefore, there is a need for postoperative adjuvant chemotherapy (POAC) after liver resection in patients with CRLM. A randomized controlled trial has shown that POAC with oral uracil-tegafur with leucovorin (UFT/LV) prolongs recurrence-free survival (RFS) after liver resection for synchronous CRLM compared with surgery alone [8]. In contrast, the guidelines of the National Comprehensive Cancer Network (NCCN) [9] and those of the European Society for Medical Oncology (ESMO) [10] suggest that an oxaliplatin-based regimen (FOLFOX or CapeOX) is preferred after synchronous colectomy and liver resection, as well as a fluoropyrimidine regimen (capecitabine or 5-FU/leucovorin).

The superiority of an oxaliplatin-based regimen over a fluoropyrimidine regimen for synchronous CRLM is uncertain. The European Organisation for Research and Treatment of Cancer (EORTC) intergroup trial 40,983 [11] demonstrated a progression-free survival (PFS) benefit with perioperative FOLFOX over surgery alone, but no effect on overall survival (OS) in resectable CRLM. However, this study did not include comparisons between an oxaliplatin-based regimen and a fluoropyrimidine regimens. Moreover, this study evaluated perioperative treatment in resectable CRLM. Therefore, currently, there is no evidence of a better clinical outcome after POAC with an oxaliplatin-based regimen over a fluoropyrimidine regimen. Therefore, this study was conducted to compare POAC with oxaliplatin-based and fluoropyrimidine regimens using propensity score (PS) matching analysis.

## Materials and methods

### Patient selection

**Fig. 1:**
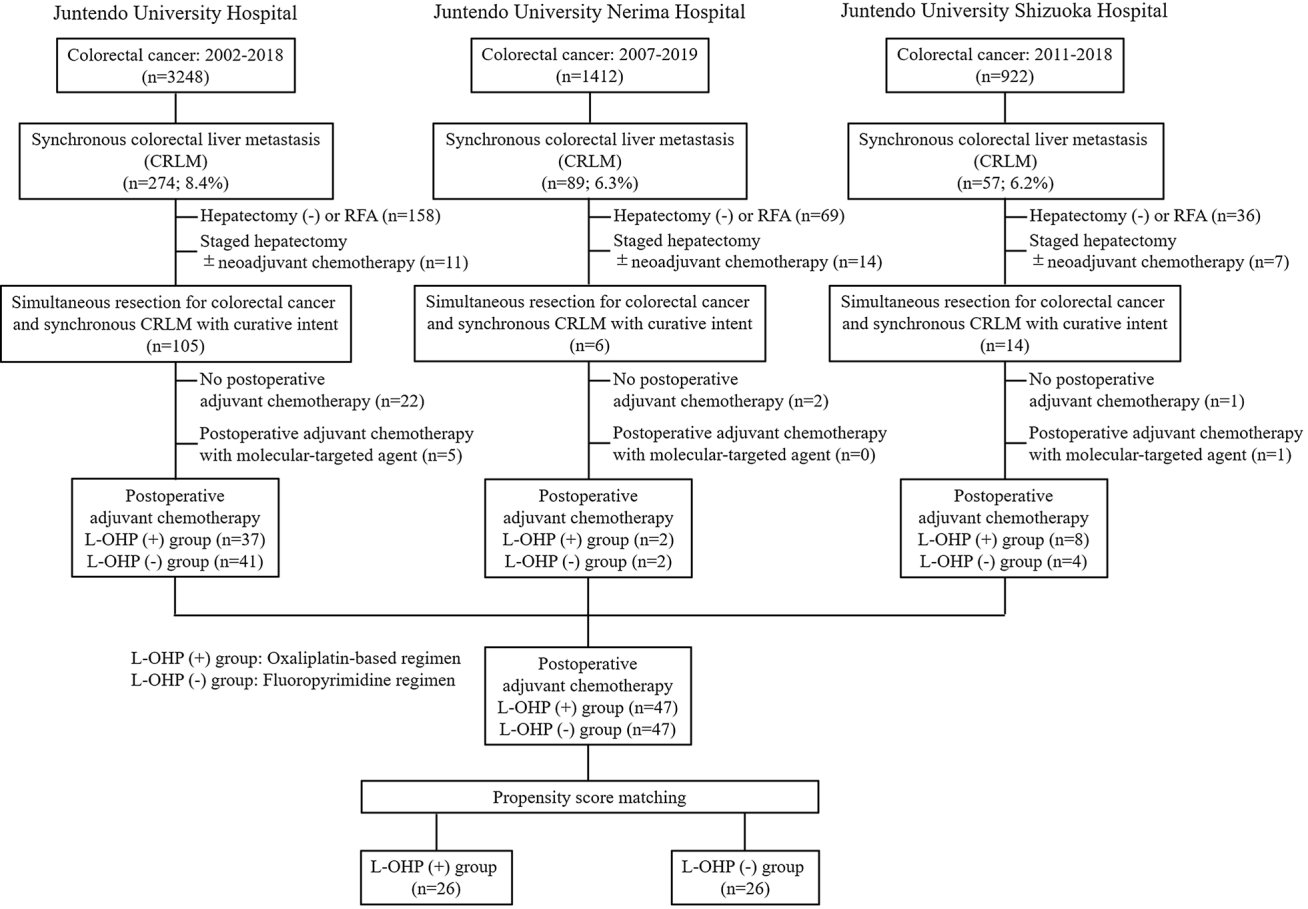
Study scheme. Of 3248, 1412 and 922 consecutive patients with colorectal cancer who were treated surgically at Juntendo University Hospital between 2002 and 2018, at Juntendo University Nerima Hospital between 2007 and 2019, and at Juntendo University Shizuoka Hospital between 2011 and 2018, respectively, 274 (8.4%), 89 (6.3%) and 57 (6.2%) presented with synchronous CRLM. Among these respective patients, 105, 6 and 14 underwent simultaneous resection for colorectal cancer and synchronous CRLM with curative intent. Finally, 78, 4 and 12 patients, who received POAC after simultaneous resection with curative intent for colorectal cancer and synchronous CRLM, were enrolled retrospectively in the study. The patients were divided into a L-OHP (+) group (POAC with an oxaliplatin-based regimen, n = 47) and a L-OHP (−) group (POAC with a fluoropyrimidine regimen, n = 47). A total of 21 patients in each group were matched in one-on-one pair PS matching analysis

The scheme of the study is shown in Fig. 1. Of 3248, 1412 and 922 consecutive patients with colorectal cancer who were treated surgically at Juntendo University Hospital between 2002 and 2018, at Juntendo University Nerima Hospital between 2007 and 2019, and at Juntendo University Shizuoka Hospital between 2011 and 2018, respectively, 274 (8.4%), 89 (6.3%) and 57 (6.2%) presented with synchronous CRLM. Among these respective patients, 105, 6 and 14 underwent simultaneous resection for colorectal cancer and synchronous CRLM with curative intent. Finally, 78, 4 and 12 patients, who received POAC after simultaneous resection with curative intent for colorectal cancer and synchronous CRLM, were enrolled retrospectively in the study. The patients were divided into a L-OHP (+) group (POAC with an oxaliplatin-based regimen, n = 47) and a L-OHP (−) group (POAC with a fluoropyrimidine regimen, n = 47). Patients who underwent POAC with molecular-targeted agents were excluded from the study because these agents were not widely used during the study period [12]. Data were collected in a retrospective review of a database and medical records. Synchronous metastases were defined as liver metastatic disease at presentation in this study. Liver resection was indicated for liver metastases for cases in which (1) curative resection was possible for a primary tumor, liver metastases and extrahepatic distant metastases; (2) there was likely to be an acceptable liver functional reserve after liver resection; and (3) the patient could tolerate surgery. The study was conducted in accordance with the Declaration of Helsinki. The study was approved by the institutional review board (IRB) of Juntendo University Hospital (No. 19-140), Juntendo University Nerima Hospital (No.2020-14) and Juntendo University Shizuoka Hospital (No. 776). The requirement for formal informed consent was waived because of the study’s retrospective design.

### Surgical strategies for colorectal cancer and synchronous CRLM

Colorectal cancer was resected with lymph node dissection at the root of the main vessels (ileocolic, middle colic, inferior mesenteric arteries and veins) with curative intent [13]. The principle of radical surgery for colorectal cancer was complete mesocolic excision (CME) or total mesorectal excision (TME) [13]. Open or laparoscopic surgery was indicated based on tumor-related factors (tumor site and extent of cancer progression) and patient-related factors (obesity, history of abdominal surgery). We note that these indications changed to some extent over the period of the study.

Preoperative evaluation using enhanced computed tomography (CT) and/or magnetic resonance imaging (MRI) was performed, as we have previously described for CRLM [14]. Occult tumors that were not detected by preoperative imaging or by inspection or palpation in laparotomy were identified by routine intraoperative ultrasound (IOUS). Relationships of tumors with vasculo-biliary structures were also shown on IOUS. Anatomical or non-anatomical IOUS-guided resection of tumors was performed to give a tumor-free margin.

### Postoperative adjuvant chemotherapy (POAC)

The POAC regimen was selected based on age and clinicopathological factors. The regimen for each case was finally decided based on a discussion between the physician and patient. POAC was started 4–8 weeks postoperatively.

### Clinicopathological analysis

Clinicopathological factors (age, gender, location in the colon or rectum, preoperative and postoperative serum carcinoembryonic antigen (CEA), differentiation of primary tumor, T classification, N classification, number of hepatic lesions, maximum diameter of hepatic lesion, presence or absence of synchronous extrahepatic metastasis, partial or anatomical liver resection, negative or positive surgical margin of a hepatic lesion), and survival were analyzed in this study. Since serum CEA has been reported to be a critical predictive factor for perioperative FOLFOX for resectable CRLM [15], it was analyzed as a categorical variable (≤ 30.0 ng/mL/ > 30.0 ng/mL, ≤ 5.0 ng/mL/ > 5.0 ng/mL), as well as a continuous variable. Postoperative serum CEA was measured once between simultaneous resection of colorectal cancer and synchronous CRLM, and the initiation of POAC. Based on International Union Against Cancer guidelines [16], negative and positive surgical margins were defined as a microscopically negative margin ≥ 1 mm, and a tumor free margin < 1 mm with microscopic tumor invasion of the resection margin, respectively.

### Follow-up

Postoperative follow-up procedures included clinical assessment and serum CEA measurements every 3 months, chest CT and abdominal ultrasonography or CT every 3–6 months.

### Propensity score (PS) matching analysis

Differences in clinicopathological severity between the L-OHP (+) and (−) groups were adjusted by PS matching analysis. The PS was estimated, and the log odds of the probability of treatment of a patient using POAC with an oxaliplatin-based regimen (L-OHP (+) group) was modeled with potential confounders of patient background factors and tumor characteristics with p < 0.05 in univariate analyses between the L-OHP (+) and (−) groups. The confounders for PS matching analysis were selected based on the factors that were mainly used to select the regimen. The c-statistic was calculated to determine the propensity model discrimination. One-on-one pair PS matching analysis with calipers < 0.05 was performed in JMP 14 (SAS Institute Inc., Cary, NC, USA).

### Statistical analysis

Recurrence-free (RFS: time from initial surgery for colorectal cancer and synchronous CRLM until first recurrence of the disease), cancer-specific (CSS: time from surgery until cancer-related death), unresectable recurrence-free (URRFS: time from surgery until first unresectable recurrence), remnant liver recurrence-free (RLRFS: time from surgery until first recurrence in the remnant liver), and extrahepatic recurrence-free (EHRFS: time from surgery until first extrahepatic recurrence) survival were determined with the Kaplan–Meier method, and significance was evaluated by univariate analyses using a log-rank test. The concept of URRFS is the same as the time to surgical failure (TSF), which could be a suitable endpoint for CRLM overall management [17]. Discrete and continuous variables were compared by Fisher exact test and Mann–Whitney U-test, respectively. JMP 14 was used for all analyses, with differences considered significant at p < 0.05. Values are shown as median (minimum–maximum).

## Results

### POAC regimens

The common regimens in the L-OHP (+) group were CapeOX (n = 25 patients, 26.6%) and FOLFOX (n = 22, 23.4%), and those in the L-OHP(-) group were UFT/LV (n = 38, 40.4%), S-1 (n = 7, 7.4%) and capecitabine (n = 2, 2.1%) (Table 1). The median numbers of cycles in each regimen in the L-OHP (+) group were 8 for CapeOX and 12 for FOLFOX. The median periods for each regimen in the L-OHP(-) group were 6 months for UFT/LV, 12 months for S-1 and 11 months for capecitabine (including discontinuation due to recurrence or adverse events).

**Table 1:** Details of the regimens of postoperative adjuvant chemotherapy (POAC)

Group	Regimen	No. of patients (%)		No. of cycles/months ^a) b)^	
L-OHP (+)	CapeOX	25	− 26.60%	8 cycles	(2–25)
	FOLFOX	22	− 23.40%	12 cycles	(1–14)
	Subtotal	47	− 50.00%		
L-OHP (−)	UFT/LV	38	− 40.40%	6 months	(1–24)
	S-1	7	− 7.40%	12 months	(2–24)
	Capecitabine	2	− 2.10%	11 months	(10, 12)
	Subtotal	47	− 50.00%		
	Total	94	− 100%		

^a^Median (minimum–maximum)

^b^Discontinuation was included due to recurrence or adverse events

The common regimens in the L-OHP (+) group were CapeOX (n = 25 patients, 26.6%) and FOLFOX (n = 22, 23.4%), and those in the L-OHP (−) group were UFT/LV (n = 38, 40.4%), S-1 (n = 7, 7.4%) and capecitabine (n = 2, 2.1%). The median numbers of cycles in each regimen in the L-OHP (+) group were 8 for CapeOX and 12 for FOLFOX. The median periods for each regimen in the L-OHP (−) group were 6 months for UFT/LV, 12 months for S-1 and 11 months for capecitabine (including discontinuation due to recurrence or adverse events)

### RFS, CSS, URRFS, RLRFS and EHRFS in the whole cohort

**Fig. 2:**
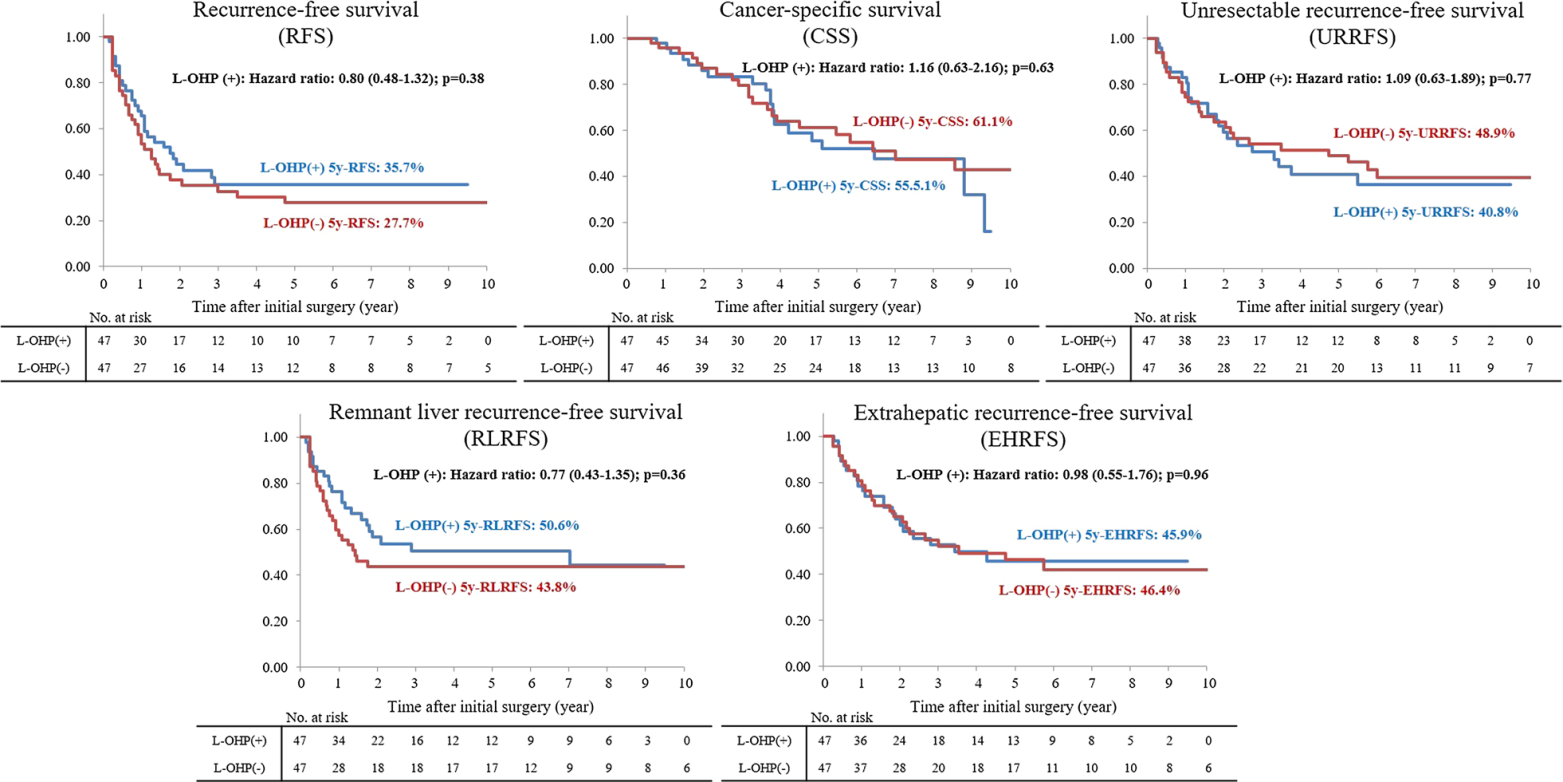
Comparisons of recurrence-free (RFS), cancer-specific (CSS), unresectable recurrence-free (URRFS), remnant liver recurrence-free (RLRFS), and extrahepatic recurrence-free (EHRFS) survival in the entire cohort. The L-OHP (+) and (−) groups had no significant differences in RFS (hazard ratio (HR): 0.80, 95% confidence interval (CI): 0.48–1.32, p = 0.38), CSS (HR 1.16, 95% CI 0.63–2.16, p = 0.63), URRFS (HR 1.09, 95% CI 0.63–1.89, p = 0.77), RLRFS (HR 0.77, 95% CI 0.43–1.35, p = 0.36), and EHRFS (HR 0.98, 95% CI 0.55–1.76, p = 0.96)

The median observation period was 64.5 months (range: 7.5–163.1 months) for recurrence-free surviving patients. The L-OHP (+) and (−) groups had no significant differences in RFS [hazard ratio (HR) 0.80, 95% confidence interval (CI) 0.48–1.32, p = 0.38], CSS (HR 1.16, 95% CI 0.63–2.16, p = 0.63), URRFS (HR: 1.09, 95% CI 0.63–1.89, p = 0.77), RLRFS (HR 0.77, 95% CI 0.43–1.35, p = 0.36), and EHRFS (HR 0.98, 95% CI 0.55–1.76, p = 0.96) (Fig. 2).

### Clinicopathological factors in the L-OHP (+) and (−) groups

Univariate analysis indicated that age, preoperative serum CEA (≤ 30.0 ng/mL/ > 30.0 ng/mL), differentiation of primary tumor (differentiated/undifferentiated), T classification (T1-3/T4), number of hepatic lesions and maximum diameter of the hepatic lesion differed significantly between the L-OHP (+) and (−) groups. Patients in the L-OHP (+) group were younger (p = 0.02); more frequently had preoperative serum CEA > 30.0 ng/mL (p = 0.04), an undifferentiated primary tumor (p = 0.03) and a T1–T3 primary tumor (p = 0.046); and had more (p = 0.04) and larger (p = 0.03) hepatic lesions (Table 2). There were no significant differences in other clinicopathological factors between the two groups.

**Table 2:** Clinicopathological factors in the L-OHP (+) and (−) groups before and after propensity score matching

Clinicopathological factors	Variables	Before matching		p-value	After matching		p-value
		L-OHP (+) (n = 47)	L-OHP (−) (n = 47)		L-OHP (+) (n = 21)	L-OHP (−) (n = 21)	
Age	Years^a^	61 (32–78)	67 (37–85)	0.02	67 (49–78)	62 (37–82)	0.28
Gender	Male	31 (66.0%)	29 (61.7%)	0.83	17 (81.0%)	15 (71.4%)	0.72
	Female	16 (34.0%)	18 (38.3%)		4 (19.0%)	6 (28.6%)	
Location	Colon	39 (83.0%)	36 (76.6%)	0.61	17 (81.0%)	15 (71.4%)	0.72
	Rectum	8 (17.0%)	11 (23.4%)		4 (19.0%)	6 (28.6%)	
Preoperative serum CEA	ng/mL^a^	38.5 (1.1–3585)	13.6 (2.0–2759)	0.14	12.0 (2.0–442.8)	20.3 (2.6–622.7)	0.62
Preoperative serum CEA	≤ 30.0 ng/mL	22 (46.8%)	33 (70.2%)	0.04	14 (66.7%)	14 (66.7%)	1.00
	> 30.0 ng/mL	25 (53.2%)	14 (29.8%)		7 (33.3%)	7 (33.3%)	
Preoperative serum CEA	≤ 5.0 ng/mL	10 (21.3%)	8 (17.0%)	0.79	7 (33.3%)	3 (14.3%)	0.28
	> 5.0 ng/mL	37 (78.7%)	39 (83.0%)		14 (66.7%)	18 (85.7%)	
Postoperative serum CEA^b^	ng/mL^a^	2.5 (0.8–115.6)	2.7 (0.4–66.3)	0.94	2.0 (0.9–115.6)	2.4 (0.4–10.9)	0.41
Postoperative serum CEA^b^	≤ 30.0 ng/mL	41 (91.1%)	43 (95.6%)	0.68	19 (95.0%)	21 (100%)	0.49
	> 30.0 ng/mL	4 (8.9%)	2 (4.4%)		1 (5.0%)	0 (0%)	
Postoperative serum CEA^b^	≤ 5.0 ng/mL	30 (66.7%)	37 (82.2%)	0.15	16 (80.0%)	19 (90.5%)	0.41
	> 5.0 ng/mL	15 (33.3%)	8 (17.8%)		4 (20.0%)	2 (9.5%)	
Differentiation of primary tumor	Differentiated	41 (87.2%)	47 (100%)	0.03	21 (100%)	21 (100%)	1.00
	Undifferentiated	6 (12.8%)	0 (0%)		0 (0%)	0 (0%)	
T classification	T1–3	37 (78.7%)	27 (57.5%)	0.046	16 (76.2%)	14 (66.7%)	0.73
	T4	10 (21.3%)	20 (42.5%)		5 (23.8%)	7 (33.3%)	
N classification	N0	15 (31.9%)	11 (23.4%)	0.49	9 (42.9%)	7 (33.3%)	0.75
	N1, 2	32 (68.1%)	36 (76.6%)		12 (57.1%)	14 (66.7%)	
Number of hepatic lesions	^a^	2 (1–21)	1 (1–10)	0.04	2 (1–5)	1 (1–6)	0.35
Maximum diameter of hepatic lesion	mm^a^	37 (8–120)	25 (5–85)	0.03	32 (8–65)	30 (8–85)	0.73
Synchronous	Absent	45 (95.7%)	46 (97.9%)	1.00	19 (90.5%)	21 (100%)	0.49
extrahepatic metastasis	Present	2 (4.3%)	1 (2.1%)		2 (9.5%)	0 (0%)	
Liver resection	Partial	29 (61.7%)	30 (63.8%)	1.00	17 (81.0%)	13 (61.9%)	0.31
	Anatomical	18 (38.3%)	17 (36.2%)		4 (19.0%)	8 (38.1%)	
Surgical margin of hepatic lesion	Negative	41 (87.2%)	40 (85.1%)	1.00	19 (90.5%)	18 (85.7%)	1.00
	Positive	6 (12.8%)	7 (14.9%)		2 (9.5%)	3 (14.3%)	

^a^Median (minimum–maximum)

^b^n = 90 (postoperative serum CEA was not available in four patients)

Univariate analysis indicated that age, preoperative serum CEA (≤ 30.0 ng/mL/ > 30.0 ng/mL), differentiation of primary tumor (differentiated/undifferentiated), T classification (T1-3/T4), number of hepatic lesions and maximum diameter of the hepatic lesion differed significantly between the L-OHP( +) and (−) groups. Patients in the L-OHP (+) group were younger (p = 0.02), more frequently showed preoperative serum CEA of higher than 30.0 ng/mL (p = 0.04), more frequently had an undifferentiated primary tumor (p = 0.03) and a T1–T3 primary tumor (p = 0.046), and had more (p = 0.04) and larger (p = 0.03) hepatic lesions. There were no significant differences in other clinicopathological factors between the two groups

The PS was estimated with potential confounders of age, preoperative serum CEA, differentiation of primary tumor, T classification, number of hepatic lesions, and maximum diameter of hepatic lesions. A total of 21 patients in each group were matched in one-on-one pair PS matching analysis. There were no significant differences in univariate analysis of clinicopathological factors between the L-OHP (+) and (−) groups after PS matching

The PS was estimated with potential confounders of age, preoperative serum CEA, differentiation of primary tumor, T classification, number of hepatic lesions, and maximum diameter of hepatic lesions. The median PS was 0.72 (0.04–1.00) in the L-OHP (+) group and 0.32 (0.04–0.77) in the L-OHP (−) group (p < 0.001). The c-statistic of 0.82 (95% CI 0.73–0.91, p < 0.001) showed satisfactory discrimination. A total of 21 patients in each group were matched in one-on-one pair PS matching analysis (Fig. 1). There were no significant differences in univariate analysis of clinicopathological factors between the L-OHP (+) and (−) groups after PS matching (Table 2).

### RFS, CSS, URRFS, RLRFS and EHRFS in the propensity-matched cohort

**Fig. 3:**
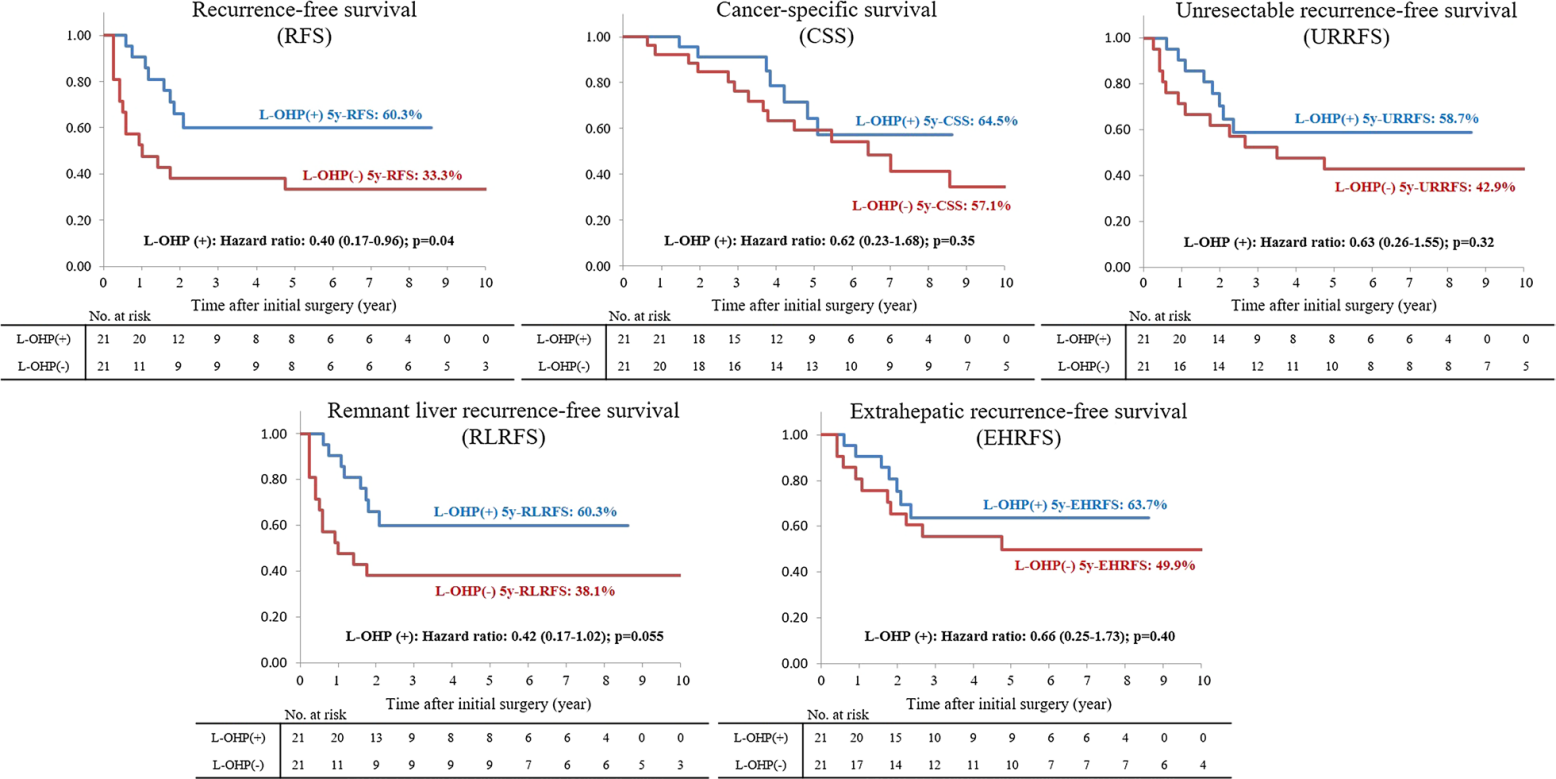
Comparisons of recurrence-free (RFS), cancer-specific (CSS), unresectable recurrence-free (URRFS), remnant liver recurrence-free (RLRFS), and extrahepatic recurrence-free (EHRFS) survival in the propensity-matched cohort. RFS was significantly better among patients in the L-OHP (+) group compared with the L-OHP (−) group (HR 0.40, 95% CI 0.17–0.96, p = 0.04). In addition, there was a trend towards better RLRFS among patients in the L-OHP (+) group compared with the L-OHP (−) group (HR 0.42, 95% CI 0.17–1.02, p = 0.055). CSS (HR: 0.62, 95% CI 0.23–1.68, p = 0.35), URRFS (HR 0.63, 95% CI 0.26–1.55, p = 0.32), and EHRFS (HR 0.66, 95% CI 0.25–1.73, p = 0.40) did not differ significantly between the two groups

RFS was significantly better among patients in the L-OHP (+) group compared with the L-OHP (−) group (HR 0.40, 95% CI 0.17–0.96, p = 0.04). In addition, there was a trend towards better RLRFS among patients in the L-OHP (+) group compared with the L-OHP (−) group (HR 0.42, 95% CI 0.17–1.02, p = 0.055). CSS (HR 0.62, 95% CI 0.23–1.68, p = 0.35), URRFS (HR 0.63, 95% CI 0.26–1.55, p = 0.32), and EHRFS (HR 0.66, 95% CI 0.25–1.73, p = 0.40) did not differ significantly between the two groups (Fig. 3).

## Discussion

Synchronous CRLM is found in about 20% of cases of colorectal cancer [12, 18]. Synchronous CRLM has less favorable cancer biology, and the 5-year survival rate after curative resection is only 39% [19]. Patients with recurrence of synchronous CRLM are thought to have had residual undetectable micrometastases when synchronous CRLM is initially resected. Therefore, use of POAC in this setting may be helpful [12, 19], but the role of POAC is still controversial.

In a study of the efficacy of POAC with an oxaliplatin-based regimen compared to a fluoropyrimidine regimen, Kim et al. [20] found that this regimen gave significantly better disease-free survival (DFS) compared to cases treated with a fluoropyrimidine regimen, but without a significant difference in OS between these groups. Hsu et al. [21] also reported that POAC with an oxaliplatin-based regimen resulted in favorable RFS compared with a fluoropyrimidine regimen, but not OS, in multivariate analysis. However, the backgrounds of the groups were not balanced, and there were trends (not significant) in the number and maximum size of synchronous CRLM between the two groups. Therefore, these differences require adjustment.

Age, preoperative serum CEA (≤ 30.0 ng/mL/ > 30.0 ng/mL), differentiation of primary tumor (differentiated/undifferentiated), T classification (T1-3/T4), and the number and maximum diameter of synchronous CRLM differed significantly between the L-OHP (+) and (−) groups in our study. Therefore, the efficacy of POAC with an oxaliplatin-based regimen was examined using PS matching analysis. The PS reflects the treatment assignment probability based on baseline covariables [22], and there is increasing use of PS methods to counter the effects of confounding factors in observational studies of the effects of treatment on outcomes [23]. There may be significant differences between patients treated with POAC with oxaliplatin-based and fluoropyrimidine regimens in clinical practice, particularly for age, tumor aggressiveness, and patient status. Therefore, we accounted for the selection bias and non-random treatment groups through use of PS matching analysis.

Baseline data in the whole cohort indicated more use of POAC with an oxaliplatin-based regimen for younger cases with more severe tumor aggressiveness, but one-on-one pair PS matching balanced these factors between the L-OHP (+) and (−) groups. We believe that this is the first use of PS matching analysis to examine the efficacy of POAC with an oxaliplatin-based regimen after simultaneous resection of colorectal cancer and synchronous CRLM. Our results indicate that the oxaliplatin-based regimen has limited efficacy in these patients. RFS was significantly better among patients in the L-OHP (+) group compared with the L-OHP (−) group. In addition, there was a trend towards better RLRFS among patients in the L-OHP (+) group compared with the L-OHP (−) group. However, there was no difference in CSS between the two groups. The absence of a difference in CSS may be because the patients in the L-OHP (−) group underwent systemic chemotherapy with an oxaliplatin-based regimen after recurrence, which may have prolonged CSS in the L-OHP (−) group. These results are similar to those in the two earlier studies [20, 21], which did not balance patient backgrounds between the groups. This indicates that cases who undergo simultaneous resection of colorectal cancer and synchronous CRLM may not require an oxaliplatin-based regimen immediately after the initial surgery. Such POAC may not be necessary until recurrence, in order to avoid oxaliplatin-related adverse events especially in elderly patients.

There are some limitations in this study. First, data were collected only for a small number of patients. Second, the POAC regimens were not constant over the study period. Third, since the oxaliplatin-based regimen was introduced more recently than the fluoropyrimidine regimen, there was a difference in study periods between the L-OHP (+) and (−) groups. This might have affected better RFS and RLRFS in the L-OHP (+) group after PS matching analysis because preoperative imaging with CT and MRI has also become more sophisticated. Fourth, data for adverse events in both regimens were not available. Finally, the RAS gene mutation status was not available in all cases, and this status affects the risk of recurrence of CRLM [24, 25].

## Conclusions

RFS was significantly better among patients in the L-OHP (+) group compared with the L-OHP (−) group. In addition, there was a trend towards better RLRFS among patients in the L-OHP (+) group compared with the L-OHP (−) group. However, there was no difference in CSS between the two groups. Therefore, the efficacy of POAC with an oxaliplatin-based regimen after simultaneous resection of colorectal cancer and synchronous CRLM is limited.

## Data Availability

The datasets used and/or analysed during the current study are available from the corresponding author on reasonable request.

## Abbreviations

CRLM: Colorectal liver metastases
POAC: Postoperative adjuvant chemotherapy
UFT/LV: Uracil-tegafur with leucovorin
RFS: Recurrence-free survival
NCCN: National Comprehensive Cancer Network
ESMO: European Society for Medical Oncology
EORTC: European Organisation for Research and Treatment of Cancer
OS: Overall survival
PS: Propensity score
L-OHP: Oxaliplatin
IRB: Institutional review board
CME: Complete mesocolic excision
TME: Total mesorectal excision
CT: Computed tomography
MRI: Magnetic resonance imaging
IOUS: Intraoperative ultrasound
CEA: Carcinoembryonic antigen
CSS: Cancer-specific survival
URRFS: Unresectable recurrence-free survival
RLRFS: Remnant liver recurrence-free survival
EHFRS: Extrahepatic recurrence-free survival
TSF: Time to surgical failure
