# Correction to: Significance of postoperative adjuvant chemotherapy with an oxaliplatin-based regimen after simultaneous curative resection for colorectal cancer and synchronous colorectal liver metastasis: a propensity score matching analysis

**DOI:** 10.1186/s12893-021-01211-5

**Published:** 2021-04-26

**Authors:** Kiichi Sugimoto, Kazuhiro Sakamoto, Yuki Ii, Kota Amemiya, Hiroyuki Sugo, Tomoaki Ito, Shinya Munakata, Makoto Takahashi, Yutaka Kojima, Yuichi Tomiki, Koichi Sato, Akio Saiura, Seiji Kawasaki

**Affiliations:** 1 Department of Coloproctological Surgery, Juntendo University Faculty of Medicine, 2‑1‑1 Hongo, Bunkyo‑ku, Tokyo, 113‑8421 Japan; 2 Department of General Surgery, Juntendo University Nerima Hospital, Tokyo, Japan; 3 Department of Surgery, Juntendo University Shizuoka Hospital, Shizuoka, Japan; 4 Department of Hepatobiliary‑Pancreatic Surgery, Juntendo University Faculty of Medicine, Tokyo, Japan

## Correction to: BMC Surg (2021) 21:188 10.1186/s12893-021-01193-4

Following the publication of the original article [1], the authors have notified us of a mistake in the Legend for Fig. 1, in the lowest column of the study scheme:

Incorrect line: L-OHP( +) group (n = 26), L-OHP( −) group (n = 26).

Corrected line: L-OHP( +) group (n = 21), L-OHP( −) group (n = 21).

The original article has been corrected.
